# A Safety Warning Algorithm Based on Axis Aligned Bounding Box Method to Prevent Onsite Accidents of Mobile Construction Machineries

**DOI:** 10.3390/s21217075

**Published:** 2021-10-25

**Authors:** Cynthia Changxin Wang, Mudan Wang, Jun Sun, Mohammad Mojtahedi

**Affiliations:** 1 School of Built Environment, University of New South Wales, Sydney, NSW 2052, Australia; cynthia.wang@unsw.edu.au (C.C.W.); m.mojtahedi@unsw.edu.au (M.M.); 2 School of Civil and Hydraulic Engineering, Huazhong University of Science and Technology, Wuhan 430074, China; sunjunym@hust.edu.cn

**Keywords:** construction machinery, accident prevention, axis aligned bounding box method, location sensing technology, safety warning algorithm

## Abstract

Mobile construction machineries are accident-prone on a dynamic construction site, as the site environment is constantly changing and continuous safety monitoring by human beings is impossible. These accidents usually happen in the form of machinery overturning or collapsing into risk areas, including the foundation pit, slopes, or soft soil area. Therefore, preventing mobile construction machineries from entering risk areas is the key. However, currently, there is a lack of practical safety management techniques to achieve this. Utilizing a wireless sensor device to collect the location information of mobile construction machineries, this research develops a safety warning algorithm to prevent the machineries moving into risk area and reduces onsite overturning or collapsing accidents. A modified axis aligned bounding box method is proposed according to the movement patterns of mobile construction machineries, and the warning algorithm is developed based on the onsite safety management regulations. The algorithm is validated in a real case simulation when machinery enters the warning zone. The simulation results showed that the overall algorithm combining the location sensing technology and the modified bounding box method could detect risk and give warnings in a timely manner. This algorithm can be implemented for the safety monitoring of mobile construction machineries in daily onsite management.

## 1. Introduction

With the increasing complexity of modern construction projects, the types and quantity of onsite construction machinery are also increasing, which brings more challenges for the safety management on construction sites. For example, in terms of housing and municipal engineering projects in China, the number of construction accidents has been increasing from 2015 to 2019, namely 442, 634, 692, 734, and 773 for each year [1]. Specifically, the 2018 and 2019 annual statistical reports of construction accidents in China show that 29.63% and 30.78% of total accidents in respective years have involved machineries, and they are from three categories: (1) construction machine and equipment injury; (2) foundation/ground collapsing accidents, which are caused by a lack of management of mobile machinery, and (3) others including vehicle injury [2,3]. Although there is no specific category for accidents related to mobile construction machineries, it is widely known that, compared to fixed construction equipment and facilities, mobile construction machineries are more frequently involved in onsite accidents.

Accidents involving construction machineries are likely to cause long project delays and large economic loss, as construction machineries are key components to keep projects on schedule and are also expensive equipment [4,5]. Some major accidents caused by mobile construction machineries which were widely reported in China, e.g., a mobile crane collapsed at the edge of a construction foundation pit in August 2017 in Harbin, China, causing one death and direct economic losses of US $0.26 million [6]. In January 2020, a construction machine collapsed at the edge of a deep foundation pit in Chongqing, China and overturned into the foundation pit, causing one death and direct economic losses of US $0.22 million [7].

Mobile construction machinery accidents usually happen at the edge of foundation pits, sloped sites, and soft soil areas, since these risks are not always visible for machine operators [8,9], leading to overturning of the mobile machinery or collapsing into soft soil. These types of accidents are often reported by various news media, and two examples are given in the references [10,11].

Although poor safety awareness and behaviour have been the focus of construction safety research in developing countries [12,13,14,15,16,17], technologies can certainly be utilized to improve onsite safety management practice [18,19]. There are many on-site risk areas for moving machinery, and accidents can only be avoided by preventing the machinery from entering their proximity [20]. There are two challenges in onsite safety management for mobile construction machineries. First, the construction site environment is dynamic and constantly changing, and therefore machine operators might be unfamiliar with the surroundings [21]. Second, safety management of construction machinery is currently mainly carried out by human monitoring and supervision and is usually inadequate and error prone [22,23]. As it is impossible for people to constantly monitor the movement of construction machinery onsite, an automated and effective way to monitor mobile machinery and issue safety warning of potentially dangerous situations is required throughout the whole construction process.

Some location acquisition and mobile communication techniques have been introduced to monitor safety of construction site, such as collecting mobility information of moving objects using GPS data [24], Bluetooth beacons for acquiring trajectory information of workers [20,25], as well as RFID and magnetic field technologies for proximity detection and alert of risky working areas [20]. These studies can be used for proximity analysis to prevent collisions, identify unauthorized access to hazardous areas, and to support the reduction of transportation accidents. However, previous works are more based on monitoring workers’ activities to avoid accidents, and an effective trajectory monitoring method focused on mobile machinery on construction sites remains lacking. This paper aims to develop a safety warning algorithm to prevent mobile construction machineries from entering risk areas, to minimize or totally avoid overturning and collapsing accidents.

## 2. Literature Review

Many previous studies focused on the collapse of foundation pits or tunnels caused by deformation of a large settlement, ineffective original reinforcement, or lack of stability of support structures, while few studies considered collapsing accidents caused by mobile machinery [26,27,28,29]. How to prevent mobile machineries from overturning/collapsing into risk areas has not been explored. There are some previous studies on overturning accidents, but they are mainly focused on those machines whose main function is lifting operations. For example, Edwards et al., [30] and Mitrev and Marinković [31] calculated the safe working load and simulated operations to enhance the excavator stability of lifting tasks. Huidong et al., [32] made a virtual prototype simulation for safety of lifting operations on mobile cranes. These studies are limited to the lifting process rather than moving status of mobile machinery on construction sites.

Proximity detection/collision warning needs to collect real-time location data [20]. There are different types of position data acquisition technologies or devices available that can be implemented on construction sites, such as GPS, sensors and RFID [33,34]. Many commercial positioning and sensing devices can be used to provide location data of targeted objects or areas [20,35,36]. The method of proximity status analysis between mobile construction machineries and risk areas can also be regarded as an anti-collision or collision detection method. Previous studies on collision detection are mainly focused on preventing collision between machineries [22,35,36,37,38], or between construction machineries and fixed construction items, or building elements [33,34] and workers on construction sites [39,40]. Ren and Wu [22] proposed a real-time automated anti-collision system to prevent collisions between a crane and fixed obstacles during the crane lifting operation process. Hwang [37] used ultra-wideband technology for the real-time monitoring of equipment operation and designed a platform with several functional modules, including real-time data acquisition, visualization, decision making, and possibility analysis of collision. Li, Gu and Jia [34] designed an anti-collision method using ultrasonic sensors to locate obstacles, and multi-sensor information fusion technologies are integrated for anti-collision warning. RFID can also be used for collision detection of construction machinery, such as hydraulic excavators and cranes [20,41]. Jo, Lee, Kim, Kim and Choi [39] designed an RFID-based warning and excavator control system to prevent collision accidents between equipment and workers. Most of these studies involved in proximity situations that focused mainly on preventing collisions between machinery and obvious obstacles. To prevent mobile machinery entering risk area, the main challenge is to detect the relationship of machinery with the identified risk areas from live onsite location data. For construction projects, using just-in-time onsite position data to give early safety warning and prevent accidents is an important area to be developed [42,43]. Based on previous published studies in this field, there is a lack of an effective algorithm developed for continuously monitoring the mobile machineries and preventing them entering risk areas on construction sites. In this study, a practical collision detection algorithm is developed which can use real-time location data of construction machinery to give safety warnings when machinery is entering onsite risk areas.

## 3. Research Methodology

The development of a safety warning algorithm has a few steps, including the establishing warning rules, developing a collision detection algorithm, collecting real-time location data and coordinating system development, as well as implementation and testing.

### 3.1. Establishing Warning Rules

Before the safety warning algorithm can be developed, warning rules need to be determined based on the onsite management regulations. Warning rules include safe distances and the classification of warning states.

#### 3.1.1. Determining Safety Distances

The safety distance is the minimum distance that should be maintained between construction machinery and the risk area. Many rules, technical specifications, and regulations have been established to regulate the behavior of workers and machinery on construction sites. For example, China’s “Administrative Regulations on the Work Safety of Construction Projects” states that warning signals must be clearly set up at the edge of holes, foundation pits, and other dangerous places [44]. China’s “Technique Specification for Safety Operation of Construction Machinery” also specifies requirements for safety distances when machineries are working around these areas, as shown in Table 1.

On a construction site, the real-time distance between a construction machinery and risky area is to be compared with the specified safety distance. The real-time distance must be bigger than the safety distance, so when machinery is getting close to a risky area, the operator can have sufficient time to brake or make a turn to avoid entering the area. To make the algorithm suitable to be used for any machinery, the maximum distance requirement for all types of machines is adopted in this research, which is 2 m, as shown in Table 1. However, this distance could be adjusted in different situations and countries when implemented in real cases. For machineries moving at a relatively high speed and when more response time is needed, this distance can be further increased.

#### 3.1.2. Classify Safety Warning States

**Figure 1:**
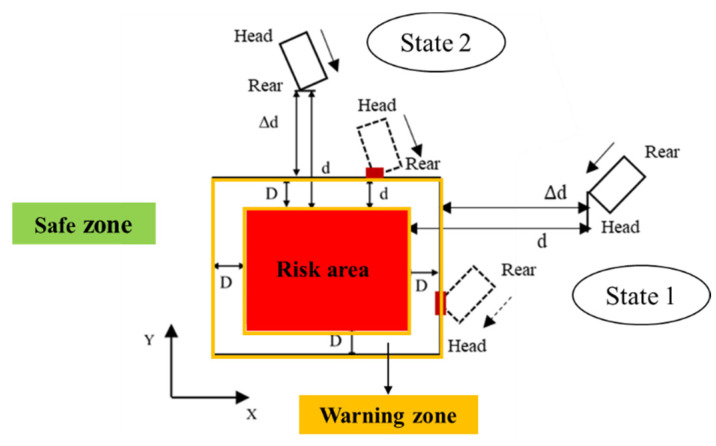
Relationship of moving machinery with risk area and warning states.

The safety distance is used to distinguish three areas (Figure 1): the safe zone, the warning zone, and the risk area. The safe zone refers to the area that the machinery can work safely in and there is no need to take any prevention actions. The warning zone refers to the area that is less than the safety distance from the risk area, as specified in site regulations, where there is a need to take actions to move away and prevent accidents happening. The risk area refers to areas, such as foundation pits, sloped areas, or soft soil, where overturning and collapsing accidents will happen if construction machinery enters. The size of a risk area can be adjusted in consideration of the slope stability and soil property. For the construction site with an unstable slopy area, the risk area should be marked bigger according to empirical data.

The movement pattern of construction machinery is examined. Construction machineries are usually large in size and can only move forward or backward in a straight or curved line. This means that either the head or rear part of the machinery will move into the risk area first. Therefore, warning signals will be triggered if the head or rear part of the machine enters the warning zone, and Figure 1 shows these two statuses: State 1 checks if there is a collision between the head of the moving machinery and the warning zone, and State 2 checks if there is a collision between the rear of the machinery and the warning zone. The distance between the nearest point of a moving machinery to the boundary of risk area is d, to the boundary of the warning zone is ∆*d*, and the safety distance adopted in this paper is D = 2 m (Figure 1).

Safe status: d > D;

Warning status: d ≤ D and ∆*d* is decreasing;

Accident happening in risk area: d ≤ 0.

According to the movement patterns of construction machineries, the two critical states are checked to trigger the warning signals.

- State 1: The head of the machine enters the warning zone

If the moving machine is going towards the warning zone, the critical state is that d = D and ∆*d* = 0, as shown in Figure 1. The head of the machine intersects with the warning zone boundary, which triggers the warning signal to be sent immediately. There is no need to conduct further detection of the rear part of the machine. Otherwise, it indicates that the head of the machine is in a safe zone, and then State 2 test is conducted to check if the rear part of the machinery intersects with the warning zone boundary.

- State 2: The rear of the machine enters the warning zone

In a similar way, if the rear of the machine intersects with the warning zone, the critical state is that d = D and ∆*d* = 0, as shown in Figure 1. The rear of the machine intersects with the warning zone boundary, which triggers the warning signal to be sent immediately. If there is no intersection, then combined with the results of the State 1 and State 2 checking, the machine is in a safe zone.

**Figure 2:**
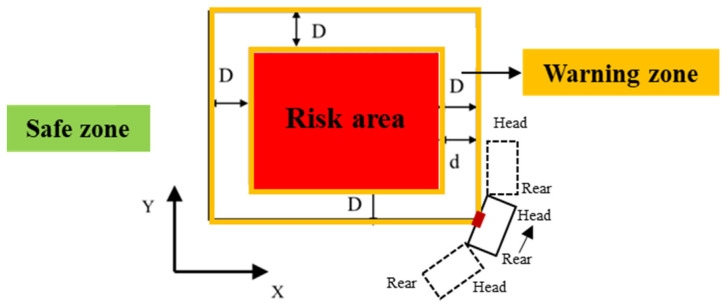
Special state when side edge of a machinery collides with warning zone boundary.

There is a third state worth some discussion, which is when the side edge of moving machinery collides with the warning zone, but the head and rear part of the machinery are in safe zone, as shown in Figure 2.

This is a special state when a machine is making a turn close to the warning zone, and the side edge of the machine collides with the corner of the warning zone. In this case, the contour of the whole machine has collided with the warning zone. However, it is still in the safe stage as both the head and rear parts of the construction machinery are located in the safe ground, and this state is of “temporary nature” and the machinery can return to a safe state where no part collides with the warning zone. This state is regarded as a safe state, but noting this state is important for the collision detection design which will be discussed in the next section.

### 3.2. Collision Detection Method

Whether machinery is at risk is based on the relative position between the head and rear of the machinery to the warning zone. From the 2D projection view of the construction site, machinery entering a warning zone can be taken as a collision problem in image processing. The design of a collision detection mechanism should consider the purpose of detection and performance of different collision methods.

#### 3.2.1. The Bounding Box Methods 

**Figure 3:**
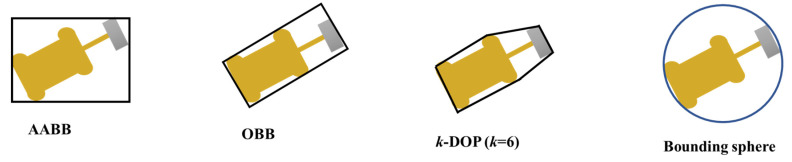
Different bounding boxes for object representation.

Collision detection is based on the principle that two non-penetrating objects cannot share the same area, and collision can be predicted by estimating if the surfaces of two non-penetrating objects are going to touch each other [47,48]. Bounding boxes are commonly used to process the intersection of objects by using simplified shapes [49,50]. Checking the intersection among bounding boxes is almost always more efficient than checking the intersection of complex objects [48,50]. Commonly used bounding boxes include the axis aligned bounding box (AABB), oriented bounding box (OBB), K-discrete orientation polyhedral (k-DOP), and bounding sphere. The representations of these methods are shown in Figure 3 [50,51,52]. Among them, AABB is the easiest to develop, as the box is bounded by the minimum and maximum coordinates of the object in each dimension [53,54,55]. The OBB bounding box is of the minimum area that covers a complex object tightly, whose coordinate system is chosen individually based on the orientation of the object [50,53]. k-DOP is a convex polytope which is bounded by spaces aligned with axes from a small, fixed set of k orientations (where k is an even number) [52,56,57]. Bounding sphere is the minimum enclosing sphere of objects and is simple and fast for intersection, as only the radiuses are required to compute the distances between objects [49,50].

Two main factors need to be considered in selecting a bounding box: computational cost and accuracy [50]. Computational cost includes the coding complexity of bounding the object and the computational intensity of collision detection [50,53]. Accuracy is usually determined by the tightness or compactness between objects and the bounding box [51,54]. In terms of computation costs, the AABB and bounding sphere are simple and fast for processing, and for continuous collision tests, sphere bounding box method can be faster than AABB [50,54]. OBB is more complex in processing as it needs to rotate with objects, which requires additional computation effort [50,52]. k-DOP is the most computationally intensive method since the coordinate system needs to rotate with objects and k times overlapping tests are required [50,51]. The k-DOP method has the highest accuracy in representing objects and the sphere box has the least accuracy in most cases [52,54]. OBB is the minimum box, so it is a more accurate representation than AABB, but it is less compact than k-DOP [52,55]. In summary, the rank of each bounding box method in terms of computational costs and compactness is given in Table 2. Each method has advantages and disadvantages, and the selection of the bounding box needs to consider the characteristics of objects and the application scenarios.

In this study, the warning zone on a construction site can be taken as a two-dimensional shape. Although a machine is a three-dimensional object, the intersection test can be carried out between the warning zone and the projection of moving machinery on the ground. For the majority of construction machines, their projection on the ground is close to a rectangle. Therefore, AABB or OBB are better options, as they are of the same shape as the objects that they represent [54,56]. As can be seen from Table 2, AABB is more efficient in the computational process, while OBB has higher accuracy. Therefore, these two methods are further considered for this study.

#### 3.2.2. A Modified Bounding Box Method

The purpose of collision detection is to identify the critical State 1 and State 2 as shown in Figure 1. First, OBB bounding box is considered, as OBB can accurately represent the whole machine for the collision test with the warning zone. However, if OBB is adopted, the special state demonstrated in Figure 2 could be detected as a collision, while the machine is actually in a safe state. Therefore, using OBB to represent the whole machine is not an effective method as it may give false alarms. For safety warnings, it is important that false alarms are avoided, as if the warning signals are not reliable, they may be ignored by onsite operators as a habit and cannot effectively prevent accidents. Therefore, the development of the bounding box needs to exclude the middle area of the machine to prevent false alarms.

**Figure 4:**
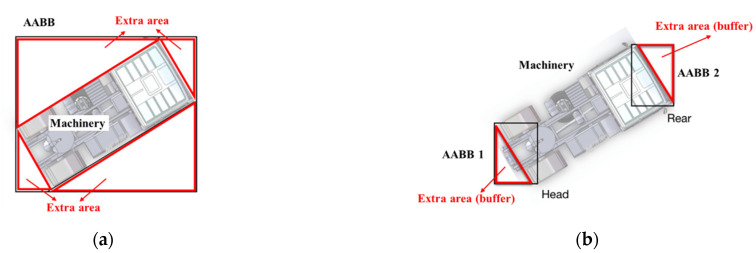
Comparison of conventional AABB and improved AABB. (**a**) Conventional AABB: extra area causing false alarm. (**b**) Improved AABB: extra area as warning buffer.

The AABB bounding box is considered due to its computational efficiency for practical purpose. The traditional way to develop AABB representing the whole machinery could result in extra areas being included, as shown in Figure 4a. Based on the states illustrated in Figure 1 and Figure 2, only the head and rear parts of the machinery need to be considered. Therefore, two AABB bounding boxes can be used to represent the front and rear parts of the machinery, respectively, as illustrated in Figure 4b. As the machine is not always parallel to the coordinate axis when it is moving, there may be extra areas of these AABB bounding boxes be included [54]. However, as the sizes of the two AABBs (front and rear) are much smaller than the original AABB representing the whole machine, these extra areas are much smaller and the errors caused by them can be ignored. In fact, these extra areas can be regarded as a small warning buffer, as these extra areas are always in the same direction as the machinery’s movement. When the extra area collides with the warning zone, it indicates the machine is moving towards the warning zone and will collide with it. Therefore, this modified AABB bounding box method not only reduces the large error areas of the original single AABB bounding box which includes the middle part of the machinery, but also enhances the effectiveness of the safety alarm with a small additional warning buffer.

As seen in Figure 4b, the minimum and maximum coordinates in abscissa and vertical axes of the two AABB bounding boxes for machinery are determined by the position data of four wheels. These position data and the position data of the risk area need to be determined and a coordination system established for collision detection.

### 3.3. Position Data Acquisition and Coordinate System Development

#### 3.3.1. Position Data Acquisition

In this study, the risk area is always simplified as a rectangle shape to represent a site area with potential risk, such as a foundation pit. The risk area is also taken as a large AABB bounding box in the collision detection, and its position is determined by the location of its four corners.

**Figure 5:**
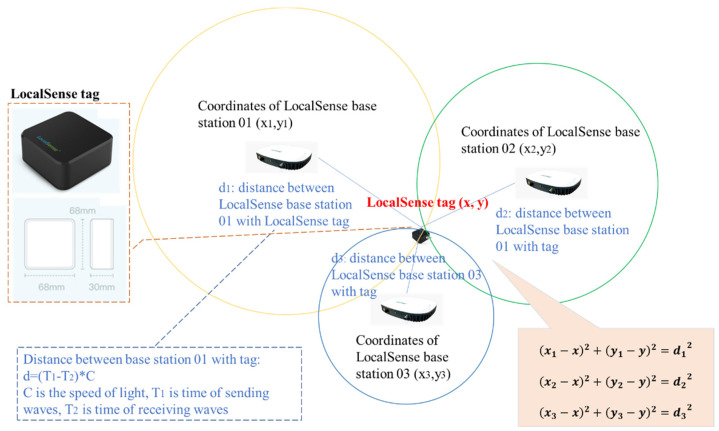
Approach of obtaining coordinates (x, y) of LocalSense tag.

To obtain the location data of the risk area and mobile machinery, a wireless positioning device needs to be adopted [37,57]. Currently, there are multiple applications in the market which can be used to collect position data [58]. LocalSense is a widely used sensor device for detecting location in China with high accuracy of 10 mm, as shown in Figure 5, and it is adopted in this research. This device has two parts: tags and base stations, and it has its own coordination system (referred to as the global coordinate system in this paper). The system can capture real-time position based on ultrawide band antennas and calculate coordinates of tags with collected data information [59,60]. The mechanism of determining the location of the LocalSense tags is illustrated in Figure 5: Two base stations can provide two possible locations of the tag (two circles have two common points), and the third station can determine the exact location (x, y) of the tag. Mathematically, the tag position (x, y) is resolved using the three equations in Figure 5: the three base stations’ coordinates are (x_1_, y_1_), (x_2_, y_2_), (x_3_, y_3_) in the global coordinate system, and d_1_, d_2_, and d_3_ are the distances between each base station and the tag, determined by the signal’s travelling time between the tag and the base stations. Therefore, three base stations can be installed on a construction site, and with four tags attached to the wheels of mobile machinery, the global coordinates H_1_(x_h1_, y_h1_), H_2_(x_h2_, y_h2_), R_1_(x_r3_, y_r3_), and R_2_(x_r4_, y_r4_) of the four wheels can be collected.

In the same way, tags are installed at the corners of the identified risk area and their coordinates can be obtained. These tags can be reused and can be moved when the risk areas change. To prevent the tags of the risk area from destroying by machinery, the tags will be removed after the coordinates of the risk area are captured by the locating device. The obtained coordinates will be recorded in the software system.

#### 3.3.2. Coordinate System of Risk Area

**Figure 6:**
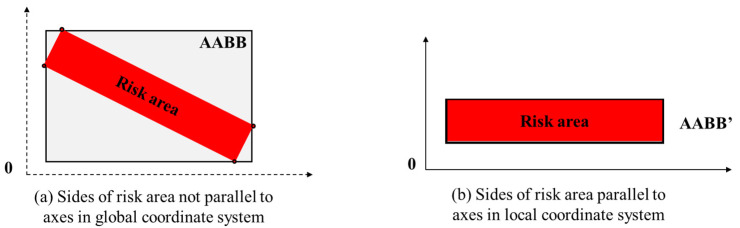
AABB development of risk area.

In most cases, the risk area is not parallel to the global coordinate system. As the usual AABB development takes the maximum coordinates of the rectangular shape, it is not an accurate representation of the risk area in the global coordinate system (Figure 6a). In this research, a local coordinate system is established for the risk area with the x and y axes of the coordinate system parallel to the sides of risk area, and the AABB is exactly the same as the risk area, which removes the errors caused by using the global system directly, as shown in Figure 6b.

**Figure 7:**
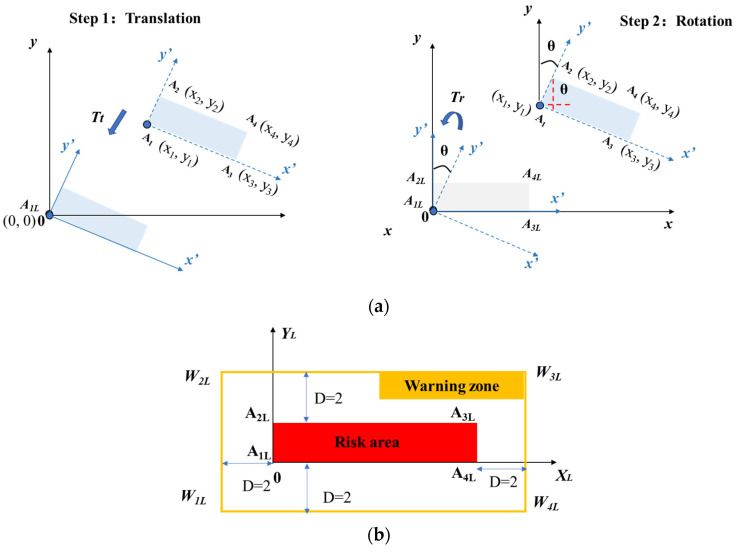
Calculation of transformation matrix T and development of local coordinate system. (**a**) Development of the T transformation matrix. (**b**) Local coordinate system of the risk area and warning zone.

Establishment of the local coordinate system also allows multiple risk areas which are not parallel to each other (and to the global system) to be processed separately and effectively. Transformation between global coordinate system and the local coordinate system of risk area is required, and it is shown in Figure 7. The transformation matrix T is used to establish a local coordinate system. The available data are four coordinates of the risk area collected by positioning application, namely A_1_ (x_1_, y_1_), A_2_ (x_2_, y_2_), A_3_ (x_3_, y_3_), A_4_ (x_4_, y_4_). The way to calculate T can be regarded as a matrix to transform coordinate *A_i_* in global system to *A_iL_* in local system, where *A_iL_* is the original point in local coordinate system.


(1) $$T=T_{t}\cdot T_{r}=[\begin{array}{ccc} 1 & 0 & 0\\ 0 & 1 & 0\\ -\left(x_{1}\right) & -\left(y_{1}\right) & 1 \end{array}]\cdot \left[\begin{array}{ccc} \cos\left(-\theta \right) & \sin\left(-\theta \right) & 0\\ -\sin\left(-\theta \right) & \cos\left(-\theta \right) & 0\\ 0 & 0 & 1 \end{array}\right]$$

(2) $$\theta =\arctan\frac{x_{2}-x_{1}}{y_{2}-y_{1}}$$

After transformation, coordinates of risk area are A_1L_ (x_1L_, y_1L_), A_2L_ (x_2L_, y_2L_), A_3L_ (x_3L_, y_3L_), A_4L_ (x_4L_, y_4L_), where A_iL_ can be calculated as:

(3) $$\left[\begin{array}{ccc} x_{iL} & y_{iL} & 1 \end{array}\right]=\left[\begin{array}{ccc} x_{i} & y_{i} & 1 \end{array}\right]\cdot T=\left[\begin{array}{ccc} x_{i} & y_{i} & 1 \end{array}\right]\cdot \left[\begin{array}{ccc} 1 & 0 & 0\\ 0 & 1 & 0\\ -\left(x_{1}\right) & -\left(y_{1}\right) & 1 \end{array}\right]\cdot \left[\begin{array}{ccc} \cos\left(-\theta \right) & \sin(-\theta ) & 0\\ -\sin\left(-\theta \right) & \cos\left(-\theta \right) & 0\\ 0 & 0 & 1 \end{array}\right],\;\left(i=1,2,3,4\right)$$

As mentioned in Section 3.1, the coordinates used for collision detection are from the warning zone rather than risk area. Therefore, coordinates of warning zone can be represented as W_1L_ (x_1L_ − 2D, y_1L_ − 2D), W_2L_ (x_2L_ − 2D, y_2L_ + 2D), W_3L_ (x_3L_ + 2D, y_3L_ + 2D), W_4L_ (x_4L_ + 2D, y_4L_ − 2D), as seen in Figure 7b).

#### 3.3.3. Coordinates of the Mobile Machinery

**Figure 8:**
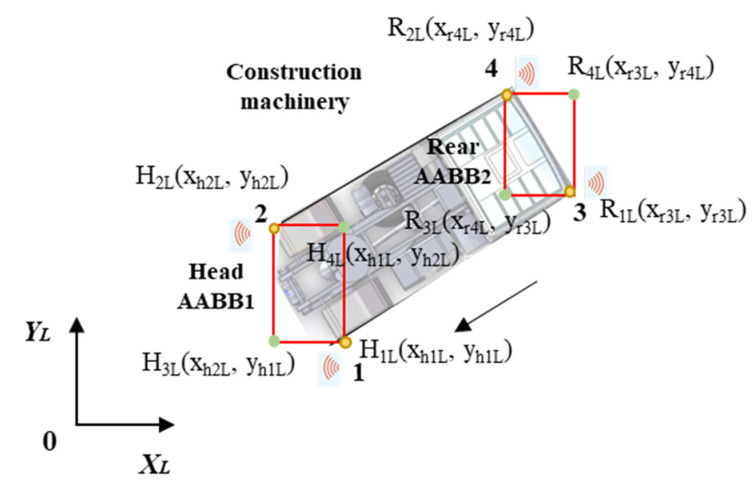
Coordinates of moving construction machinery and AABB boxes in local coordinate system.

As presented in Section 3.2.2, coordinates of the head and rear parts of mobile machinery are required to establish modified AABB bounding box. Therefore, four wheels are marked as points 1, 2, 3, and 4, as shown in Figure 8. The coordinates of the two head wheels are H_1_(x_h1_, y_h1_) and H_2_(x_h2_, y_h2_), and coordinates of rear wheels are R_1_(x_r3_, y_r3_) and R_2_(x_r4_, y_r4_). Currently coordinates of machinery are also collected in global system, which should be transformed to the local system as developed in Figure 8. By using transformation matrix T to get the coordinates in the same way, the local coordinates of machinery are H_1L_(x_h1L_, y_h1L_), H_2L_(x_h2L_, y_h2L_), R_1L_(x_r3L_, y_r3L_), R_2L_(x_r4L_, y_r4L_). Based on the four coordinates, the modified AABB method can be developed to represent the machinery as designed. Therefore, in the local system, the other two coordinates of head AABB_1_ are H_3L_(x_h2L_, y_h1L_) and H_4L_(x_h1L_, y_h2L_), and the other two are R_3L_(x_r3L_, y_r4L_) and R_4L_(x_r4L_, y_r3L_) for rear AABB_2_. All coordinates of the modified AABB are obtained, as shown in Figure 8. The models of the warning zone and construction machinery are established in the same local coordinate system.

### 3.4. Implementation of Safety Warning Algorithm

#### 3.4.1. Proximity Detection/Collision Warning Analysis

**Figure 9:**
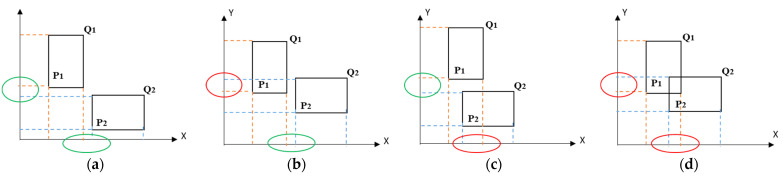
Identification of collision between two AABB: (**a**) No collision (**b**) No collision (**c**) No collision (**d**) Collision.

The critical process is to conduct proximity detection/collision warning analysis using collected location data. After the coordinate system development of the pre-identified warning zone and machinery in a local system, proximity detection is used to check if the AABB representing the machinery (head or rear) intersect with the AABB representing the warning zone on either the X or Y axes. The algorithm of intersection tests is given as follows:

for (i = 0; i < 2; i++)

if L_1i_ > H_2i_ || L_2i_ > H_1i_

return 0;

else return 1;

Where the minimum and maximum vertices of the two AABBs are P_1_(L_10_,L_11_), Q_1_(H_10_,H_11_), and P_2_(L_20_,L_21_), Q_2_(H_20_,H_21_), as illustrated in Figure 9. Based on the above algorithm, if it returns “1”, it means the two boxes intersect or are colliding. Otherwise, if the algorithm returns “0”, it means there is no collision. There are four possible relationships between the two AABB bounding boxes. If the two AABBs do not intersect with each other on both axes, there is no collision between them, as marked in green circles in Figure 9a. If there is an intersection on either of the axes with no intersection on the other axis, the two AABB still do not intersect with each other, as seen in Figure 9b,c. If the two AABB intersect with each other on both axes, there is collision between them as marked in red circles in Figure 9d.

This proximity detection will be adopted to check if the modified AABB bounding boxes (representing the head and rear of a mobile machinery) collide with the warning zone. As the machinery is constantly moving, a continuous checking mechanism needs to be established.

#### 3.4.2. Warning Algorithm Implementation

**Figure 10:**
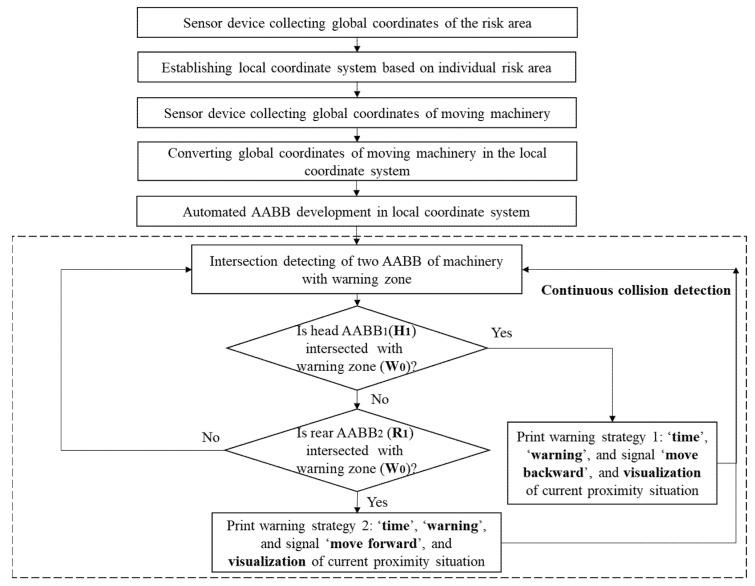
System design of safety warning algorithm.

Both head and rear AABB bounding boxes of machinery need to be tested using the proximity detection with the warning zone (W_0_). For practical onsite operation, most of the time the machine is in a state of moving forward, so the front wheels are more likely than the rear wheels to move into the warning zone (W_0_). Therefore, it is reasonable to test collision of the head AABB_1_ (H_1_) first followed by the rear AABB_2_ (R_1_). The complete process for implementing the collision detection is given in Figure 10. The warning signal can be given instantly based on the developed algorithm.

## 4. Case Simulation and Validation 

### 4.1. Case Background

**Figure 11:**
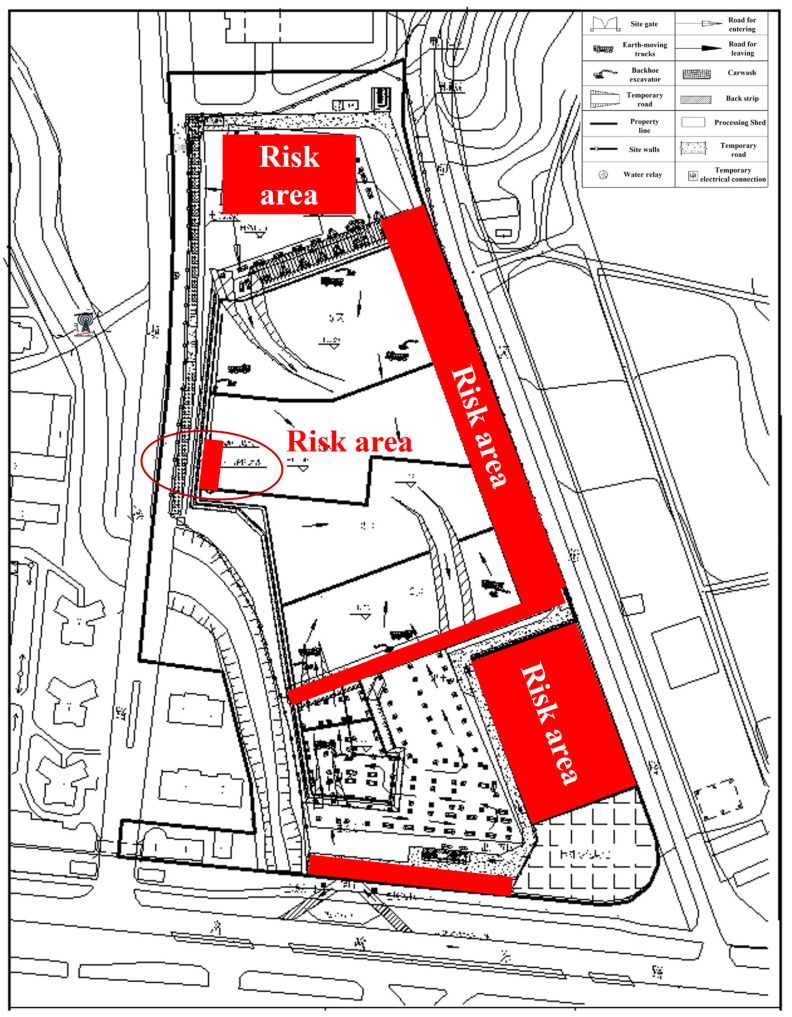
Case example of potential risk area for mobile machinery and arrangement of positioning application.

A real case simulation is carried out to validate the proposed algorithm. Figure 11 shows a construction site layout. The site is complex with different construction areas, and some foundation pits are under excavation, which are potential risk areas for onsite mobile machineries, and they are presented as red rectangles in Figure 11. Some risk areas are not exactly in the shape of a rectangle. However, they are simplified to the closest rectangular areas for an easier process. In this study, a relatively small risk area, marked in a red circle in Figure 11, is selected for case simulation to validate the algorithm. The dimension of the area can be determined directly from the coordinates captured by the tags installed at the four corners of the risk area.

**Figure 12:**
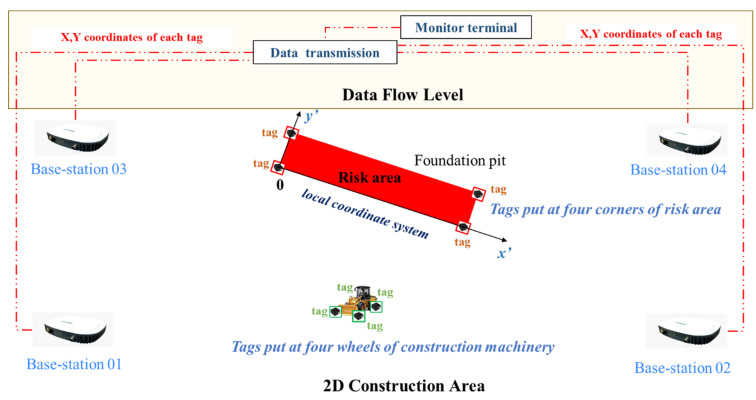
Layout of base-stations and tags on construction site (not proportional to site dimensions).

As mentioned in Section 3.3.3, three base-stations could determine the position of a tag. In this case simulation, a total of four base stations are installed, and the system will use the closest three base station data to calculate the tags’ location. The layout of the base-stations and the tags for collecting coordinates are shown in Figure 12 (diagram is not in proportion with the real distance on site). The base-stations could obtain x, y coordinates of these tags in global coordinate system. Then, these coordinate data are transmitted to a monitor terminal, where the proposed algorithm is installed. These coordinates are the input data of the proposed safety warning algorithm. The data flow is also shown in Figure 12, which demonstrates how tags on the risk area and machinery in the construction site are linked with the proposed safety warning algorithm.

### 4.2. Model Development and Collision Test Results

The location data collected by the tags installed at the four corners of the risk area are expressed in the global coordination system as A_1_ (22.62, 27.86), A_2_ (26.72, 34.96), A_3_ (44.35, 15.31), and A_4_ (48.45, 22.41). As discussed in Section 3.3.2, transformation matrix T is applied to convert these data into the local coordinate system, which is computed as:

(4) $$T=\left[\begin{array}{ccc} 1 & 0 & 0\\ 0 & 1 & 0\\ -22.62 & -27.86 & 1 \end{array}\right]\cdot \left[\begin{array}{ccc} 0.866 & 0.5 & 0\\ -0.5 & 0.866 & 0\\ 0 & 0 & 1 \end{array}\right]=\left[\begin{array}{ccc} 0.866 & 0.5 & 0\\ -0.5 & 0.866 & 0\\ -5.659 & -35.442 & 1 \end{array}\right]$$

The local coordinates are then obtained as A_1L_ (0, 0), A_2L_ (0.0, 8.2), A_3L_ (25.1, 8.2), and A_4L_ (25.1, 0). Therefore, the risk area is of the dimension 8.2 × 25.1 m. The warning zone is identified as W_1L_ (−2.0, −2.0), W_2L_ (−2.0, 10.2), W_3L_ (27.1, 10.2), W_4L_ (27.1, −2.0). Four tags are installed on the wheels of machinery. Tag 1, tag 2, tag 3, and tag 4 are used for continuous location data collection of H1, H2, R1, and R2 respectively. The global coordinate data are collected real-time at every 0.5 s and are input into the safety warning algorithm simultaneously. Multiple rounds of simulation and data collection were carried out, which validated the effectiveness and accuracy of the proposed algorithm. In this paper, two key datasets are reported, one is data collected while moving forward and another is collected while moving backward. Machinery is simulated to be moving on the construction site, starting from about 20 m away from the risk area. A wheel loader was taken as the machinery in the simulation, and its approximate dimension is 1.98 × 2.82 m. In some reported research, the working speed of wheel loader is about 5 km/h (around 1.39 m/s) on a construction site [61,62]. Therefore, the simulation speed of moving forward is set as 1.5 m/s, and the speed of moving backward is set as 1 m/s. Table 3 shows the continuously collected location data (every 0.5 s) of the four tags on the wheel loader. These data are the input data of the safety warning algorithm.

**Figure 13:**
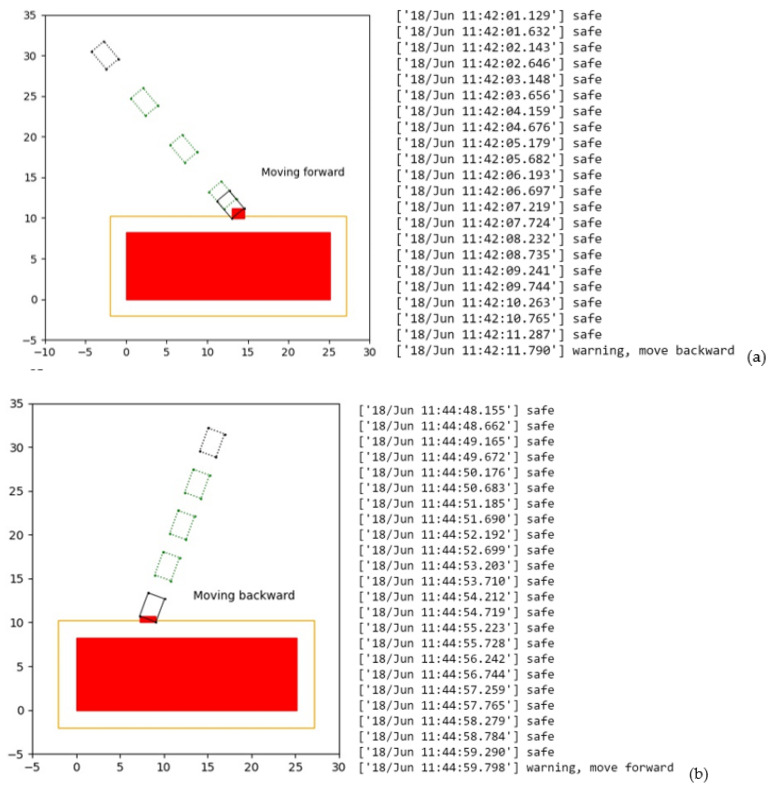
Moving trajectory and system output of algorithm: (**a**) moving forward (**b**) moving backward.

In terms of data collected while moving forward, the starting position of machinery as the location data collected by the 4 tags is H_1_ (36.63, 53.92), H_2_ (34.68, 53.58), R_1_ (36.14, 56.70), and R_2_ (34.19, 56.36). After transformation to the local coordinate system, the coordinates are H_1L_ (−0.89, 29.57), H_2L_ (−2.41, 28.29), R_1L_ (−2.71, 31.73), and R_2L_ (−4.23, 30.46). The boxes in dotted line in Figure 13a represent the moving path of the machinery. The black box represents the machinery at the starting position. Similarly, the starting position data collected while moving backward are H_1′_ (51.74, 48.18), H_2′_ (53.02, 46.67), R_1′_ (49.58, 46.37), and R_2′_ (50.86, 44.86), and the corresponding local coordinates are H_1L′_ (15.06, 32.16), H_2L′_ (16.92, 31.48), R_1L′_ (14.09, 29.51), and R_2L′_ (15.96, 28.83), as shown in Figure 13b.

The next step is the collision detection test, which will also be carried out every 0.5 s. In the safety warning algorithm, the improved AABB of machinery will be automatically created in the local coordinate system, as shown in Figure 8. The continuous collision detection was carried out. As shown in Figure 13a, at the time of 11:42:11.790′, collision was detected, and the head bounding box was displayed in red colour and warning is given. The warning signal instructs the operator of the machinery to move backwards in a timely manner. A similar simulation was carried out while the machinery was moving backward, as shown in Figure 13b. Collision was detected at the time of 11:44:59.798′. The rear AABB was displayed in red and a warning given.

These two simulations demonstrate the safety warning signals are triggered successfully and instructions are released to the machinery operator to avoid an accident.

## 5. Discussions and Conclusions

The above case simulation represents typical situations for construction machinery moving around a risk area. The proposed algorithm could continuously process input location data of moving machinery and release safety warning signals to machinery operators when the machinery enters the warning zone.

In this simulation, the safe distance between the risk area and the warning zone boundary is set as 2 m, this distance is used according to the construction site regulations in China as listed in Table 1. Considering the fact that a machine operator needs to have some response time, and when machinery is moving at a speed of 1–1.5 m/s (about 3.6−5.4 km/hour), 2 m only provides less than 2 s of response time. Therefore, it is suggested that in real construction practice, this distance is to be increased according to the machinery’s speed. When this algorithm is used in other countries or in a specific situation, this distance can also be adjusted to provide appropriate response time to the machine operator.

The collision detection was carried out every 0.5 s in this simulation, and as the computation process is efficient and almost instant, this frequency can also be adjusted according to the speed of the machinery to meet the requirement of onsite monitoring in real projects.

This study proposed a safety warning algorithm for moving construction machineries to prevent them from entering risk areas to avoid overturning and collapsing accidents. The designed algorithm and the continuous processing of the collected location data by sensor devices offer a reliable and practical means for the automated onsite monitoring of construction machineries. There are two major contributions of this proposed algorithm.

Firstly, for improving computation efficiency and reducing false alarm, a modified axis aligned bounding box (AABB) method is proposed. AABB is a relatively simple method. However, using two AABBs to represent the head and rear part of a construction machinery respectively is a novel approach. The error caused by the simplification is minimized, and the small extra space of the two AABBs serves as warning buffer, which increases the sensitivity of the warning algorithm. The modified AABB method also eliminates the possibility of the false warning caused by the turning machinery at the corner of the warning zone. This method is suitable for monitoring onsite construction machineries as it can make a flexible representation of machinery for collision detection analysis compared with other bounding box methods, and therefore improves its reliability in capturing actual situations of onsite construction machineries with an efficient computational approach.

Secondly, the proposed algorithm could be conveniently integrated with various real-time location sensing techniques and use their own coordinate system to collect location data. It then converts the location data into a local coordinate system established for each risk area. The use of a local coordinate system enables that each risk area is accurately represented by a standard AABB bounding box and ensures the computation accuracy when multiple risk areas are included. The proposed algorithm provides a practical method for continuous monitoring of construction mobile machineries.

The simulation results demonstrate that the proposed safety warning algorithm works effectively in detecting risks and can release timely warning signals. It also requires very little data space for data storage, and is of very low computational cost. The warning signals can be triggered by using wireless communication technologies, such as Wifi and Internet of Things (IoT), and the details of implementing these technologies are reported in other studies [63,64,65].

Construction site safety management is an important aspect of an efficient overall site layout planning [66]. The limitation of this algorithm is that the risk area identified in this case needs to be simplified as a rectangular shape, while there are some risk areas in construction sites that are in circular or other irregular shapes, and some machineries could also be of a different shape, such as excavators in the form of a bounding sphere. Therefore, in the future, a more flexible bounding box development method could be proposed for risk areas and machineries of other shapes, and this could support a more efficient and accurate safety status check for different mobile machineries working close to all types of risk areas. Moreover, this research only considers proximity detection between machineries and risk areas. Thus, future research could address monitoring the relative positions of multiple machineries and preventing collision between them.

## Figures and Tables

**Table 1:** Safety distances for moving construction machinery from onsite regulations in China.

Type	Requirement of Safety Distance	Reference
Bulldozer	Keep away from deep ditch, foundation pit or steep slope area for at least 2 m	[45]
Towed scraper	Distance between two scrapers working together shall be no less than 2 m	[45]
	The distance from edge of new embankment slope shall be no less than 1 m	[45]
Wheel loader	The distance from the edge of slope, trench and pit should be more than 1.5 m when unloading	[45]
Concrete spreader	The distance from other equipment should not be less than 0.6 m	[45]
Others	Moving machinery from edge of foundation pit shall be no less than 2 m	[46]

**Table 2:** Ranking of different bounding box method in terms of computation cost and compactness [50,51,52,54,55].

Type	Computation Cost	Compactness
AABB	2	3
OBB	4	2
k-DOP	3	1
Bounding Sphere	1	4

Note: 1 represents the best and 4 represents the worst.

**Table 3:** Global coordinate datasets of four tags of machinery.

	Tag1_x	Tag1_y	Tag2_x	Tag2_y	Tag3_x	Tag3_y	Tag4_x	Tag4_y
**Data** **(Moving** **Forward)**	36.63	53.92	34.68	53.58	36.14	56.70	34.19	56.36
	36.76	53.18	34.81	52.84	36.27	55.96	34.32	55.62
	36.89	52.45	34.94	52.10	36.40	55.22	34.45	54.88
	37.02	51.71	35.07	51.36	36.53	54.48	34.58	54.14
	37.15	50.97	35.20	50.63	36.66	53.75	34.71	53.40
	37.28	50.23	35.33	49.89	36.79	53.01	34.84	52.66
	37.41	49.49	35.46	49.15	36.92	52.27	34.97	51.93
	…	…	…	…	…	…	…	…
**Data** **(Moving Bacward)**	51.74	48.18	53.02	46.67	49.58	46.37	50.86	44.86
	51.36	47.86	52.63	46.35	49.20	46.05	50.47	44.53
	50.98	47.54	52.25	46.03	48.82	45.73	50.09	44.21
	50.59	47.22	51.87	45.70	48.43	45.41	49.71	43.89
	50.21	46.90	51.48	45.38	48.05	45.09	49.32	43.57
	49.83	46.58	51.10	45.06	47.67	44.77	48.94	43.25
	49.45	46.26	50.72	44.74	47.29	44.44	48.56	42.93
	…	…	…	…	…	…	…	

## Data Availability

The data presented in this study are available on request from the corresponding author.
